# miR-18a-5p Is Involved in the Developmental Origin of Prostate Cancer in Maternally Malnourished Offspring Rats: A DOHaD Approach

**DOI:** 10.3390/ijms232314855

**Published:** 2022-11-28

**Authors:** Sergio Alexandre Alcantara Santos, Luiz Marcos Frediani Portela, Ana Carolina Lima Camargo, Flavia Bessi Constantino, Ketlin Thassiani Colombelli, Matheus Naia Fioretto, Renato Mattos, Bruno Evaristo de Almeida Fantinatti, Michela Alessandra Denti, Silvano Piazza, Sérgio Luis Felisbino, Elena Zambrano, Luis Antonio Justulin

**Affiliations:** 1 Department of Structural and Functional Biology, Institute of Biosciences, Sao Paulo State University (UNESP), Unesp Botucatu, Botucatu 18618-689, SP, Brazil; 2 Cancer Signaling and Epigenetics Program, Fox Chase Cancer Center, Philadelphia, PA 19111, USA; 3 Department of Cellular, Computational and Integrative Biology—CIBIO, University of Trento, 38123 Trento, Italy; 4 Departamento de Biología de la Reproducción, Instituto Nacional de Ciencias Médicas y Nutrición, Salvador Zubirán, Mexico City 14080, Mexico

**Keywords:** Developmental Origins of Health and Disease (DOHaD), aging, prostate cancer, epigenetics, miR-18a-5p/P4HB network

## Abstract

The Developmental Origins of Health and Disease (DOHaD) concept correlates early life exposure to stressor conditions with the increased incidence of non-communicable chronic diseases, including prostate cancer (PCa), throughout the life span. However, the molecular mechanisms involved in this process remain poorly understood. In this study, the deregulation of two miRNAs (rno-miR-18a-5p and rno-miR-345-3p) was described in the ventral prostate VP of old rats born to dams fed with a low protein diet (LPD) (6% protein in the diet) during gestational and lactational periods. Integrative analysis of the (VP) transcriptomic and proteomic data revealed changes in the expression profile of 14 identified predicted targets of these two DE miRNAs, which enriched terms related to post-translational protein modification, metabolism of proteins, protein processing in endoplasmic reticulum, phosphonate and phosphinate metabolism, the calnexin/calreticulin cycle, metabolic pathways, N-glycan trimming in the ER and the calnexin/calreticulin cycle, hedgehog ligand biogenesis, the ER-phagosome pathway, detoxification of reactive oxygen species, antigenprocessing-cross presentation, RAB geranylgeranylation, collagen formation, glutathione metabolism, the metabolism of xenobiotics by cytochrome P450, and platinum drug resistance. RT-qPCR validated the deregulation of the miR-18a-5p/P4HB (prolyl 4-hydroxylase subunit beta) network in the VP of older offspring as well as in the PNT-2 cells transfected with mimic miR-18a-5p. Functional in vitro studies revealed a potential modulation of estrogen receptor α (ESR1) by miR-18a-5p in PNT-2 cells, which was also confirmed in the VP of older offspring. An imbalance of the testosterone/estrogen ratio was also observed in the offspring rats born to dams fed with an LPD. In conclusion, deregulation of the miR-18a-5p/P4HB network can contribute to the developmental origins of prostate cancer in maternally malnourished offspring, highlighting the need for improving maternal healthcare during critical windows of vulnerability early in life.

## 1. Introduction

Advances in public healthcare policy have resulted in an improvement in longevity worldwide. However, the increase in the older population has been accompanied by a rise in chronic diseases which affect the demand for aged care services [1,2].

Over the past decades, early life exposure to unfavorable environments has been associated with alterations in the offspring’s developmental biology, shaping health and disease trajectories across the lifespan [3,4,5]. David Barker was the first researcher to propose the “fetal origin of adult diseases” hypothesis [6], which states that environmental exposure to adverse conditions during the vulnerable window of development may program lifelong outcomes in descendants. Several years later, the Developmental Origins of Health and Diseases (DOHaD) expanded the Barker hypothesis.

Despite the challenge to confirm the long-lasting effects of developmental exposure on human health, tragic historical conflicts have provided important data on this issue. As such, the “Dutch hunger winter”, a period at the end of World War II, exposed almost 4.5 million people to famine, including pregnant women and newborns. Several studies pointed to an increased incidence of cardiometabolic disease, diabetes, and schizophrenia in individuals born during this period [7,8,9]. In addition, experimental and epidemiological studies also support the DOHAD concept and point out that exposure to adverse environmental conditions during early life impacts not only the incidence of non-communicable chronic diseases but also triggers the carcinogenesis process, including breast and prostate cancer, in descendants [10,11,12,13,14,15]. In this regard, Elias et al. (2004) [16] found that girls (2 to 9 years old) exposed to famine during the Dutch hunger winter had a higher incidence of breast cancer. Additionally, Keinan-Boker et al. (2009) [11] described a higher risk of PCa in Jewish men exposed early in life to starvation and stress during the Holocaust.

Using rodent models, our research group has demonstrated that maternal LPD impairs prostate development in young male offspring and increased the incidence of prostate disorders, including PCa, in older rats [12,17,18,19,20]. However, the molecular mechanisms involved in the developmental origin of PCa are still lacking.

Accumulating evidence suggests that epigenetic factors are the main mechanistic framework involved in the offspring’s response to maternal malnutrition [21,22,23]. Among epigenetic markers, miRNAs have been described as a family of non-coding small RNAs that function in modulating gene expression at the transcriptional and posttranscriptional levels [24]. Although changes in miRNAs expression profile have been described as a key regulator of the early life origin of hepatic disturbances [25], insulin resistance [26], and kidney diseases [27], the potential effect of maternal malnutrition on modulation of miRNAs expression profile with long-lasting consequences for carcinogenesis has not yet been evaluated.

Considering the clinical relevance of PCa worldwide (GLOBOCAN), studies that aim to identify new potential environmental risk factors associated with the etiology of PCa should be helpful [28]. In the current translational study, we combined next-generation sequencing (NGS) with bioinformatics tools to identify the global expression profile of miRNAs/mRNAs networks altered by maternal malnutrition in the offspring ventral prostate (VP), which can be involved in the developmental origins of PCa in older offspring.

## 2. Results

### 2.1. Biometric and Metabolic Parameters of Pregnant and Offspring Rats

At the end of the gestational period, pregnant rats fed a low-protein diet presented a reduced body weight, even though there was no difference in energy intake observed between the CTR and GLLP groups. At birth (PND1), male and female offspring from the GLLP group had a reduced body weight compared to the CTR group. The reduction persisted until weaning PND21 (Table 1). There was no change in the number of pups per litter and the male/female ratio per litter. At PND21 and 540, the body weight was reduced in male offspring from the GLLP group compared to the CTR group (Table 1). The VP absolute and relative weights were reduced in male offspring from the GLLP group at PND21, while no differences in this parameter were observed at the PND540 compared to the CTR group (Table 1). We observed the increase in serum levels of testosterone and estrogen in the GLLP group at PND21, while at PND540 we observed reduced levels of testosterone with an increase in estrogen. A reduction of 17β-hydroxy-4-androstene-3-one (testosterone) was observed in the offspring from the GLLP group, while 17β-estradiol was increased in the GLLP group compared to the CTR group (Table 1).

### 2.2. Identification of Prostate Carcinogenesis in Offspring Submitted to Maternal Malnutrition

**Figure 1:**
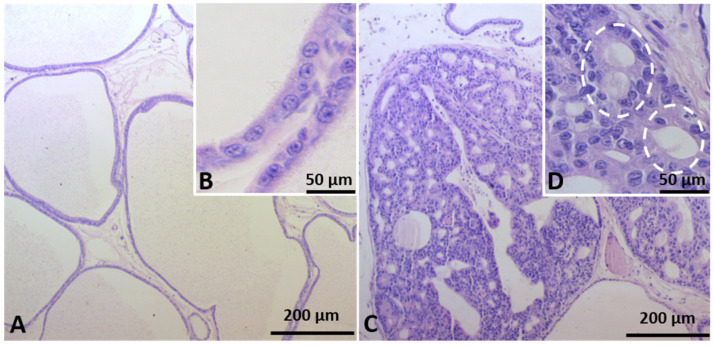
Representative histological sections of the VP lobes from the CTR (**A**,**B**) and GLLP (**C**,**D**) groups at PND 540 stained with hematoxylin/eosin (HE). In the CTR group, the VP histology resembles the structural characteristic of older VP with normal glandular and stromal compartments. In the GLLP group, the glandular lumen was filled with a dysplastic cell mass with microacinar structures (dashed circle). Scale bar: 200 µm (**A**,**C**) and 50 µm (**B**,**D**). CTR: Control; GLLP: Gestational and Lactation Low Protein.

In the CTR group, the VP showed normal morphology of the glandular and stromal compartments (Figure 1A). Interestingly, carcinoma in situ was detected in old offspring from the GLLP group. Histologically, carcinoma in situ was characterized by the presence of dysplastic cell mass into the glandular lumen and development of microacinar structures (Figure 1C,D).

### 2.3. Multiomic Analysis Identified Molecular Markers Associated with Prostate Carcinogenesis in the VP of Maternally Malnourished Offspring

**Figure 2:**
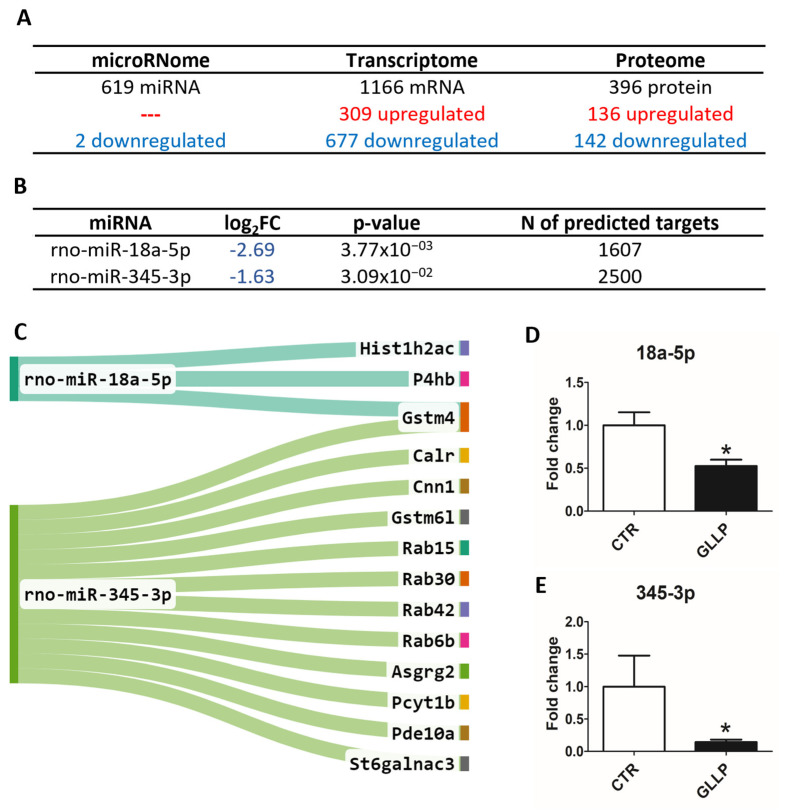
(**A**) Representative image of the differential expression of the multiomic data in the offspring VP from the GLLP group compared to the CTR group at PND 540 (for detailed data, see Supplementary Tables S1–S3). Data of microRNome and transcriptome (n = 5 from the CTR group and n = 4 from the GLLP group. Data of proteome (n = 3 for both the CTR and GLLP groups). (**B**) *Differentially expressed (DE)* miRNAs identified between the GLLP and the CTR groups. The criteria used to identify DE miRNAs were Log_2_ Fold Change ≥ |+0.4| ≤ |−0.4| and *p* < 0.05 and base mean > 20. The prediction analysis was performed on the miRWalk 3.0 tool. (**C**) Integrative analyzes using predicted targets of the DE miRNA identified in the VP transcriptome and proteome data from older offspring rats submitted to maternal malnutrition. D and E: RT-qPCR for experimental validation of the DE miRNA miR-18a-5p (**D**) and miR-345-3p (**E**) in the offspring VP. Asterisks mean the statistical difference with *p* < 0.05. CTR: Control; GLLP: Gestational and Lactation Low Protein.

Global gene and protein expression analyses by sequencing and mass spectrometry identified a total of 619 miRNAs, 1166 mRNAs, and 396 proteins in the VP samples at PND 540 (the global protein expression was accessed from a previously published paper from our group [19]). Of these, two deregulated (downregulated) miRNAs, 986 deregulated mRNAs (309 up and 667 downregulated), and 278 deregulated proteins (136 up and 142 downregulated) were observed in the GLLP group compared to the CTR group (Figure 2A; for detailed data, see Supplementary Tables S1–S3). The in silico target prediction analysis for two DE miRNAs identified a total of 1607 targets for rno-miR-18a-5p and 2500 targets for rno-miR-345-3p (Figure 2B, Supplementary Table S5). In integrative analysis, considering the opposite expression profile of downregulated miRNAs and upregulated targets (mRNAs and proteins), we identified 3 and 12 predicted targets potentially regulated by rno-miR-18a-5p and rno-miR-345-3p, respectively. The other four DE mRNAs were predicted to be regulated by rno-miR-345-3p (Figure 2C). In order to validate the miRNA sequencing for further functional analysis, the RT-qPCR confirmed the downregulation of rno-miR-18a-5p and rno-miR-345-3p in the GLLP group compared to the CTR group (Figure 2D,E).

### 2.4. Enrichment of Ontological Terms Related to DE miRNAs-Networks

**Figure 3:**
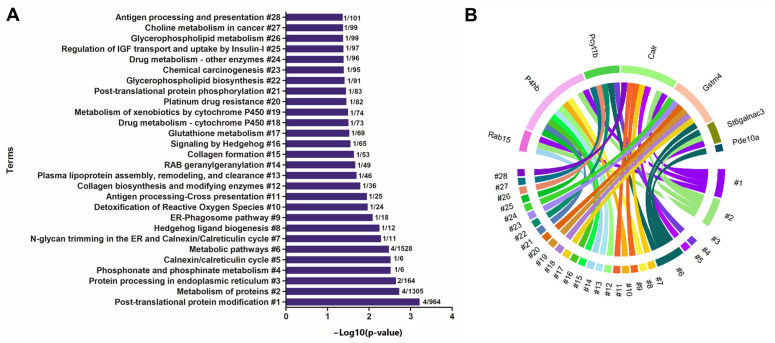
(**A**) Ontological enrichment terms by KOBAs 3.0. All data were expressed as −Log_10_ (*p*-value). The numbers in the bar graph identify the input number of predicted targets that enriched each term. (**B**) Circus plot graphic identifying the association of deregulated targets involved in each enriched term.

For enrichment analysis, the predicted targets (mRNAs and proteins) of the rno-miR-18a-5p and rno-miR0345-3 were used. The most enriched terms were related to post-translational protein modification, metabolism of proteins, protein processing in endoplasmic reticulum, phosphonate and phosphinate metabolism, calnexin/calreticulin cycle, metabolic pathways, N-glycan trimming in the ER and calnexin/calreticulin cycle, hedgehog ligand biogenesis, ER-phagosome pathway, detoxification of reactive oxygen species, antigen processing-cross presentation, collagen biosynthesis and modifying enzymes, plasma lipoprotein assembly, remodeling, and clearance, RAB geranylgeranylation, collagen formation, signaling by hedgehog, glutathione metabolism, drug metabolism—cytochrome P450, metabolism of xenobiotics by cytochrome P450, platinum drug resistance, post-translational protein phosphorylation, glycerophospholipid biosynthesis, chemical carcinogenesis, drug metabolism—other enzymes, regulation of insulin-like growth factor (IGF) transport and uptake by insulin-like growth factor binding proteins (IGFBPs), glycerophospholipid metabolism, choline metabolism in cancer, antigen processing and presentation (Figure 3A). Figure 3B identified the association between deregulated targets with each enriched term.

### 2.5. Effects of Transient Transfection of Human PNT-2 Cells on miR-18a-5p Expression and Wound Healing Capacity

**Figure 4:**
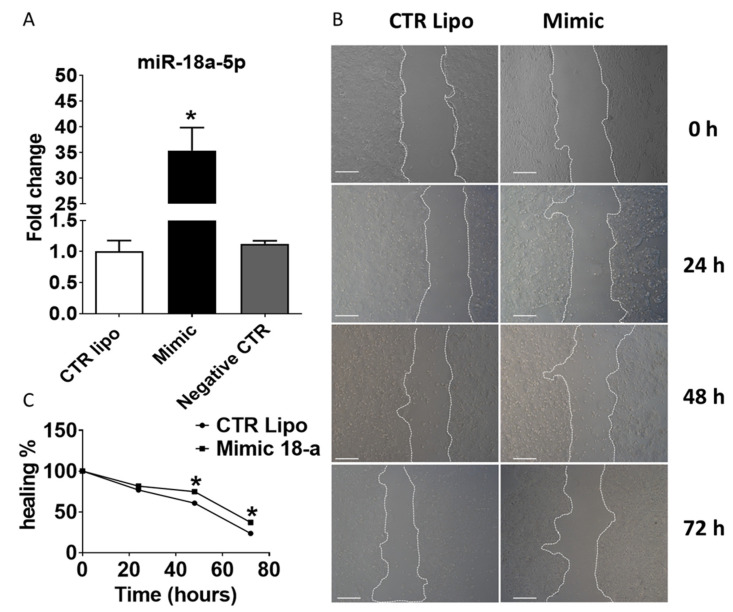
(**A**) Expression profile of miR-18a-5p in PNT-2 cells from the Control Lipo, Mimic, and Negative CTR groups after 24 h of treatment. (**B**). Representative wound healing assay images after 24, 48, and 72 h. (**C**). Cellular wound closure after the transfection of PNT-2 cells with lipofectamine and mimic miR-18a-5p after 0, 24, 48, and 72 h post-wound. Mimic group: cells treated with mimic miR-18a-5p. In vitro studies were conducted in three independent experiments performed in triplicate. Data are expressed as mean ± SD. * means a statistical difference between experimental groups with *p* < 0.05. Scale bar: 200 µm. CTR: Control.

Of the two DE miRNAs validated by RT-qPCR, the miR-18a-5p was selected to perform in vitro functional validations. The miR-18a-5p expression was increased in human PNT-2 cells transiently transfected with mimic miR-18a-5p compared to CTR Lipo and Negative Control groups (Figure 4A). The wound healing assay (an indirect method to evaluate mechanisms of cell migration and proliferation) showed that miR-18a-5p transfected cells promoted a delay in wound healing potential after 48 and 72 h of treatment (Figure 4B,C).

### 2.6. In Vitro Modulation of miR-18a-5p Confirms the Results Observed in the VP of Older Rats

**Figure 5:**
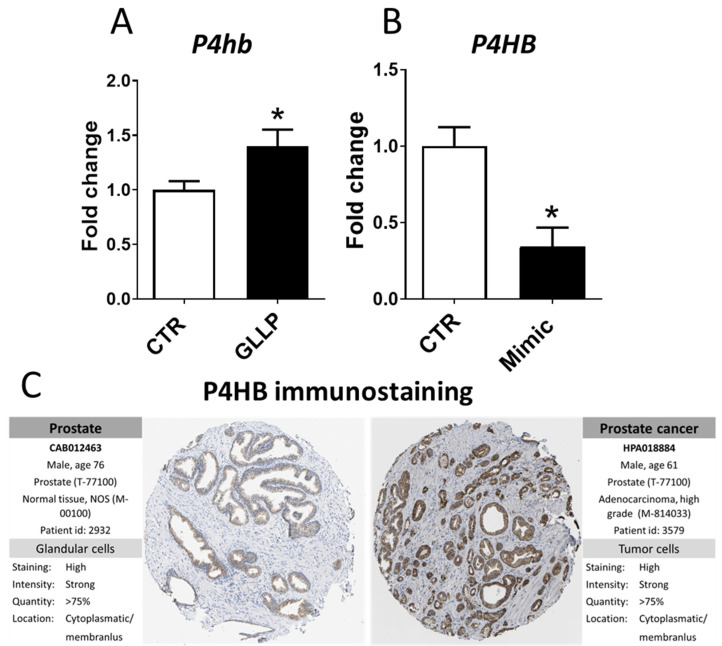
*P4hb* gene expression in the ventral prostate (**A**) of offspring rats from the CTR and GLLP groups at PND 540. (**B**) P4HB gene expression in the PNT-2 human prostate cells from the CTR and transfected with mimic miR-18a-5p groups. (**C**) P4HB immunostaining in normal and PRAD tissues obtained from The Human Protein Atlas database (https://proteinatlas.org/ (accessed on 4 May 2021)). Data are expressed as mean ± SD. * means a statistical difference between experimental groups with *p* < 0.05. CTR: Control group; GLLP: Gestational and Lactational Low Protein diet; VP: ventral prostate; PRAD: prostate adenocarcinoma.

Among the three predicted targets of miR-18a-5p (*P4HB*, *HIST1H2AC*, and *GSTM4*), the RT-qPCR analysis confirmed the upregulation of the *P4hb* in offspring VP from the GLLP group, in which rno-miR-18a-5p was downregulated compared to the CTR group (Figure 5A). Conversely, in vitro experiments showed the downregulation of *P4HB* in the PNT-2 cells transfected with mimic miR-18a-5 (Figure 5B). Interestingly, in silico analysis demonstrated intense immunostaining for *P4HB* in patients with PCa compared to the normal prostate samples (Figure 5C).

### 2.7. Potential Modulation of the Estrogen Receptors by miR-18a-5p in the Prostate

**Figure 6:**
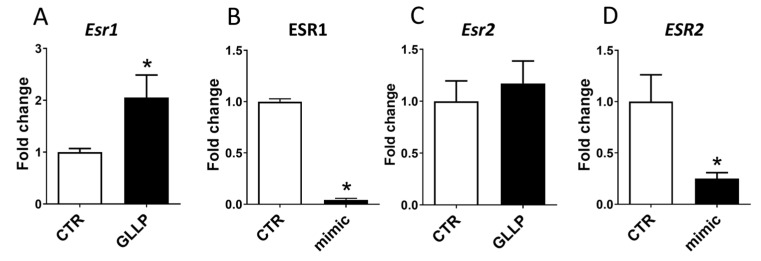
Esr1 (**A**) and Esr2 (**C**) gene expression profiles in the offspring VP from the CTR and GLLP groups at PND 540. Esr1 (**B**) and Esr2 (**D**) gene expression profiles in the PNT-2 human prostate cells from CTR and transfected with mimic miR-18a-5p. Data are expressed as mean ± SD. * means a statistical difference between experimental groups with *p* < 0.05.

Considering the key role of the estrogen receptors during prostate development and tumorigenesis, we evaluated the potential role of *miR-18a-5p* in modulating the expression profile of the estrogen receptor alpha (*ESR1*) and estrogen receptor beta (*ESR2*) in older rats submitted to maternal malnutrition. The expression of *Esr1* was increased in the VP of older rats from the GLLP group compared to the CTR group (Figure 6A), while there is no statistical difference in the (*Esr2*) expression (Figure 6C). In vitro experiments demonstrated a reduction of both *ESR1* and *ESR2* expression in PNT-2 cells transfected with miR-18a-5p mimic (Figure 6B,D).

## 3. Discussion

Over the past decades, the DOHaD concept has been consolidated, pointing out maternal malnutrition as an important risk factor for the increasing incidence of non-communicable chronic diseases in offspring, including breast and prostate cancer (PCa) [11,12,19,29,30]. Although the initial doubt to link early-life exposure to environmental stressors with long-term consequences for PCa, experimental data have confirmed that gestational and early postnatal exposure to estrogen or endocrine chemical disruptors, such as phthalates or BPA, can trigger prostate developmental biology, with last longing consequence for PCa with aging [31,32]. Such a condition was described in the African American population, whose mothers present higher levels of estrogen during pregnancy compared to Caucasian women [33], and the sons are at high risk for PCa. In one of the few opportunities to evaluate the role of early life exposure to adverse conditions on human prostate carcinogenesis, Keinan-Boker et al. (2009) [11] demonstrated that Jewish men exposed to famine and stress during early life at the Holocaust are at high risk of PCa with aging. Experimental data also demonstrated that offspring exposed to maternal malnutrition were at high risk to develop PCa with aging [12,20].

Although the molecular mechanisms related to the developmental origin of PCa remained to be elucidated, Santos et al. (2020) [19] identified the deregulation of important signaling pathways commonly altered in the prostate of both young and older offspring rats exposed to maternal LPD. The most enriched signaling pathways in both young and older rat offspring were connected to estrogenic signaling pathways, endoplasmic reticulum activities, energy metabolism, and molecular sensors of protein folding and Ca^2+^ homeostasis, according to the proteomic profile. Overall, our findings showed that maternal protein restriction changes prostate biology early in life, predisposing to a higher incidence of prostate diseases with aging.

Over the past decades, there is substantial evidence supporting epigenetics as a key molecular mechanism for developmental programming, which can lead to phenotypic adaptation during adult life. The most classical epigenetic markers involved in the modulation of gene expression are related to DNA methylation, histone modifications, and regulation by non-coding RNA. Among non-coding RNA, miRNAs have been recognized as one of the major regulatory gene families during both normal development and carcinogenesis [34]. It has been demonstrated that miRNAs can act as both tumor suppressors [35,36,37], as well oncogenic miRNAs [36,38,39] in several types of cancers.

To give further insights into the potential epigenetic modulation of gene expression in maternally malnourished offspring, RNAseq analysis was performed in the VP of offspring rats which developed carcinoma in situ with aging, as described by Santos et al. (2019) [12]. We identified two deregulated miRNAs (rno-miR-18a-5p and rno-miR-345-3p) in the VP of older rats exposed to maternal LPD. Of these, miR-18a-5p was selected for further functional validations due to its deregulation in several types of cancers, including osteosarcoma [40], prostate cancer [41], ovarian cancer [42], intestinal cancer [43], gastric cancer [44], colorectal cancer [45], nasopharynx cancer [46], lung cancer [47], glioblastoma [48], breast cancer [49], pancreatic cancer [50], and esophageal adenocarcinoma [51]. Moreover, rno-miR-18a-5p shares the same nucleotide sequence with humans (Supplementary Table S9), which allows further in vitro validation using human prostate cell lines.

Although there are published data describing the oncogenic [52] role of miR18a-5p, other studies have suggested a key tumor suppressor [53,54] function of this miRNA in several types of cancers, including ovarian cancers [54]. As such, in vitro overexpression of miR-18a-5p improved overall survival in an animal model of lung cancer by increasing radiosensitivity of lung cells and inhibiting the development of A549 xenografts after radiation treatment [55]. Furthermore, overexpression of miR-18a-5p in ovarian epithelial cells induces apoptosis, cell cycle arrest, and decrease cancer growth in vivo through directly targeting cancer signaling pathways, such as tumor protein 53 (TP53), TP53 regulated inhibitor of apoptosis 1 (TRIAP1), and inositol phosphate multikinase (IPMK) [56]. Thus, the downregulation of miR-18a-5p in the VP of older rats can be associated with prostate carcinogenesis in maternally malnourished old offspring rats.

mRNA-18a is one of the most conserved and multifunctional miRNAs in the miR-17~92 cluster, and although frequently overexpressed in malignant tumors, miR-18a has a dual functional role in either promoting or inhibiting oncogenesis in different human cancers. This characteristic has been attributed probably to the different developmental stages, genetic background, cell origins, and tumorigenic mechanisms [57,58]. Although differences in the miR-18a expression in cancers, our multiomic as well as functional validation data highlighted the deregulation of miR-18a-5p in the VP of maternally malnourished offspring rats which developed prostate cancer with aging.

Among predicted targets of miR-18a-5p identified in the integrative analysis (*Gstm4*, *Hist1h2ac*, *P4hb*), we confirmed by RT-qPCR that Prolyl 4-hydroxylase beta (*P4hb*) was upregulated in the VP of older offspring from the GLLP group, taking into account the opposite regulation of miR-18a-5p-predicted target. *P4hb* is a chaperone that works on the reparation of misfolded proteins due to endoplasmic reticulum (ER) stress [59]. Interestingly, these data matched our ontological term enrichment data which identified enriched terms related to post-translational protein modification, metabolism of proteins, protein processing in the endoplasmic reticulum, post-translational protein phosphorylation in the prostatic cells, pointing out a potential dysfunction of the endoplasmic reticulum in the offspring VP submitted to the maternal malnutrition. Upregulation of *P4HB* has been associated with carcinogenesis and tumor progression in several cancer types, including renal cell carcinoma, gastric cancer, clear cell, and colon cancer [60,61,62]. *P4HB* inhibition has been also linked to chemosensitivity in glioblastoma multiforme via the endoplasmic reticulum stress response pathways [63,64]. The upregulation of P4hb has already been identified by Santos et al. (2020) [19] in older offspring rats from the GLLP group. Strong immunostaining for P4hb was also observed in the human PCa, as demonstrated by the HPA dataset. To give further insights into the potential modulation of *P4hb* by miR-18a-5p, we transfected PNT-2 cells with mimic miR-18a-5p and we observed a reduction of *P4hb* mRNA in these cells, highlighting the potential modulation of *P4hb* expression by miR-18a-5p in prostate cells. Overall, these data highlighted the potential role of the miR-18a-5p/P4hb network in prostate cancer development in older offspring exposed to maternal malnutrition.

It is noteworthy that the prostate is highly sensitive to estrogen or estrogenic compounds during the developmental period [13,65,66] and exposure to elevated levels of estrogen can lead to increased incidence of prostate diseases with aging [10,12,19,33,67,68,69,70]. The cellular response to estrogen response is primarily mediated by ESR1 and ESR2, and changes in the expression profile of these receptors are related to the increased incidence of prostatic disorders, including PCa, with aging [71,72]. In our study, the increase in estrogen levels was accompanied by the upregulation of *Esr1* in offspring from the GLLP group. Interestingly, the PNT-2 cells treated with mimic miR-18a-5p demonstrated a reduction in the expression profile of both ERS1 and ERS2. Although estrogen receptors were not identified as predicted target miR-18a-5p, a negative correlation between miR-18a-5p and ESR1 expression was demonstrated in breast tumor cells and hepatocellular cancer [73,74], indicating a potential interaction of miR-18a-5p/ESR1. Considering the key role of estrogen in prostate biology [75], we also demonstrated the modulation of ESR1 and ESR2 in the PNT-2 cells transfected with mimic miR-18a-5p/ESR1, suggesting the involvement of miR-18a-5p/ESR1 in modulating the estrogen signaling pathway in prostate cells. Taken together, these data demonstrated the involvement ofmiR-18a-5p/ESR1 in deregulating key molecular mechanisms involved in prostate carcinogenesis in maternally malnourished old offspring rats.

## 4. Material and Methods

### 4.1. Animals

Naive adult (90 days of age) females (n = 36) and males (n = 12) Sprague Dawley rats were used. The animals were kept under a controlled temperature (22–25 °C), relative humidity (55%), and a photoperiod (12 h/12 h), with free access to water and food. Breeding proceeded overnight in a harem configuration (1 male to 3 females). After determination of pregnancy through detection of spermatozoa in the vaginal smear (considered gestational day 1—GD1), pregnant rats were distributed in the Control group (CTR, n = 18), comprised of pregnant rats fed with a normal protein diet (17% protein) during the gestation and lactation, and the Gestational and Lactational Low Protein group (GLLP, n = 18), comprised of pregnant rats fed with a low protein diet (6% protein) during the same periods. The diets followed the AIN-93 standards described by Reeves et al. (1993) [76] and were provided by PragSoluções (PragSoluções, Jaú, SP, Brazil). The diets were previously used in other studies [12,19,20]. All diets were isocaloric and normosodic and were prepared as recommended by the American Institute of Nutrition (AIN 93-G). The metabolic energy intake of the diet was calculated using the parameters established by Sadowska and Rygieska (2019) [77].

Litters were reduced to eight pups (four males and four females) on postnatal day (PND) 1 to maximize lactation performance. Dam and offspring biometric parameters were measured. One male offspring from each litter was euthanized by an overdose of ketamine and xylazine, followed by decapitation at PND 21 and 540 (n = 18/group from each age). Blood and ventral prostate (VP) were collected and processed as described below. The procedures were approved by the Biosciences Institute/UNESP Ethics Committee for Animal Experimentation (Protocol #573).

### 4.2. Hormonal Analysis

Blood samples from male offspring (n = 8/group) were centrifuged (4000 RPM for 20 min), and estrogen (17β estradiol, Monobind^®^, Lake Forest, CA 4925-300, USA, sensitivity: 8.2 pg/mL) and testosterone (17β-hydroxy-4-androstene-3-one, Monobind^®^, CA 3725-300A, USA, sensitivity: 0.038 ng/mL) were quantify using the sera offspring. An ELISA plate reader (Epoch™, Biotek Instruments, Winooski, VT, USA) was used for the quantification of these hormones in 96-well plates following the datasheet protocol.

### 4.3. Ventral Prostate Histopathology

At PND 540, samples of VP lobes from the CTR and GLLP groups (n = 12/group) were fixed in Methacarn [78], diaphanized in xylene, and embedded in Paraplast (Sigma, St. Louis, MO, USA). For the general morphological study, histological slices (5 µm) were stained with Hematoxylin/Eosin. The slides were evaluated using the image analyzer Leica Q-win software and a Leica DMLB 80 microscope connected to a Leica DC300FX (Version 3 for Windows).

### 4.4. RNA Purification, Library Construction, and Sequencing

In order to evaluate the effects of maternal malnutrition on the offspring VP miRNAs expression profile, the total RNA of the VP samples from the CTR group (n = 5) and the GLLP (n = 4) group were extracted using Trizol following the datasheet guidelines (Thermo Fisher, Waltham, MA, USA). The purified RNA was stored at −80 °C in DNase/RNase-free distilled water. The Nano Vue Plus spectrophotometer (GE Healthcare, Chicago, IL, USA) was used to verify the quality of RNA extraction and quantification, and the RNA Integrity Number (RIN) was determined by analyzing microfluidic ribosomal RNAs using the Agilent 2100 Bioanalyzer system (Santa Clara, CA, USA) [79]. Only samples with a RIN of 8 or above were used. The RNA was sequenced in a HiSeq2500 platform (Illumina, San Diego, CA, USA). For rRNA depletion, an aliquot of total unfractionated RNA was provided for a library building, and Ribo-Zero sequencing was employed during library preparation. The TruSeq Standard mRNA Sample Preparation Kit (Illumina) was used to purify messenger RNA and build libraries from total RNA, according to the manufacturer’s instructions. Small non-coding (Snc) RNA libraries were prepared by TruSeq Small RNA Library Prep Kit (Illumina, CA, USA) following the manufacturer’s specifications.

### 4.5. miRNA Sequencing Analysis

After sequencing, the miRNA raw reads quality control was carried out by FASTQ files that were pre-processed by adapter trimming and quality filtering based on the Phred quality score (≥20). Furthermore, the preprocessed sequence reads were aligned to the RattusNovegicus genome (Rnor_6.0) using the STAR package (Spliced Transcripts Alignment to a Reference), aligner, and annotated miRNA species quantified based on genomic loci, to generate the count matrix. The read count data were normalized, and the DESeq package was used to perform the differential expression analysis [80]. All these steps were performed using the OASIS Platform. The fold change between GLLP and CTR groups was calculated for each miRNA. In the Gene Expression Omnibus (GEO) database, raw Illumina miRNA-Seq FASTQ files can be found with an Accession No. GSE180674. Cut-off criteria for determination of differentially expressed (DE) miRNA were |Log2 FC| ≥ |+0.4| ≤ |−0.4|, *p*-value < 0.05, and base mean > 20 (Supplementary Table S2).

### 4.6. mRNA Sequencing Analysis

To give further insights into the relationship between the miRNAs-mRNAs networks, we reanalyzed the transcriptomic data of the VP from CTR (n = 5) and GLLP (n = 4) groups previously generated by our research group. These data were extracted from the Gene Expression Omnibus (GEO) database [81], as raw Illumina mRNA-Seq FASTQ files, and can be found with Accession No. GSE180673.

The raw data were analyzed in terms of reading quality through FastQC software (https://www.bioinformatics.babraham.ac.uk/projects/fastqc/ (accessed on 6 July 2021)) [82] to evaluate the reads and adapter low-quality. Subsequently, the single-end reads were trimmed using the Trimmomatic software (v. 0.36) and default parameters [83]. After processing by read-quality media, the remaining reads were aligned against the reference genome of Rattus Norvegicus (Genome assembly: Rnor_6.0GCA_000001895.4) obtained through the Ensembl (http://useast.ensembl.org/index.html (accessed on 6 July 2021)) through the software STAR (v. 2.5.1a) [84]. The resulting files were processed through the Feature Counts software [85] to obtain read counts and Cufflinks to obtain fragments per kilobase of transcript per million (FPKM), for specificity analysis of clustering [86].

The edgeR Bioconductor package in R^38^ was used to calculate the differential expression of RNA-seq data. DE genes between experimental groups were determined by applying the statistical cutoff of Log_2_ Fold Change ≥ |+0.66| ≤ |−0.66| and the *p*-value < 0.05 (Supplementary Table S3).

### 4.7. Prediction, Filtering, and Enrichment Analyses of the DE miRNAs’ Predicted Targets

To give further insights into the epigenetic mechanisms involved in the VP response to maternal malnutrition, the DE miRNAs identified in the VP were used to perform target prediction analyses using the miRWalk 3.0 tool (http://mirwalk.umm.uni-heidelberg.de/ (accessed on 7 July 2021)) for the Rattus norvegicus dataset (Supplementary Table S5). As described above, the transcriptomic data were downloaded from the GEO database (GSE180673). Moreover, in order to explore the potential role of the DE miRNAs in modulating the VP proteomic profile, the data of the VP global protein expression from CTR (n = 3) and GLLP (n = 3) groups were accessed from a previously published paper from our group [19]. Proteins were considered differentially expressed when *p* < 0.05 for downregulated proteins and *p* > 0.95 for upregulated proteins (Supplementary Table S6). For target prediction, we consider the opposite expression profile of miRNA and mRNAs or proteins (Supplementary Table S7).

The targets-miRNAs identified in the integrative analysis were submitted to the enrichment analysis of molecular pathways using the KOBAS (http://kobas.cbi.pku.edu.cn/anno_iden.php (accessed on 7 July 2021)) and the Enrichr (https://maayanlab.cloud/Enrichr/ (accessed on 7 July 2021)) platforms. The cut-off criterion for significance was *p*-value < 0.05 (Supplementary Table S8).

### 4.8. Validation of Selected miRNA-mRNA Networks in the Offspring VP by RT-qPCR

To validate the expression of the selected miRNA and the predicted target mRNAs, in the VP samples of older offspring rats at PND 540, the total RNA was extracted from VP samples (n = 6/group) in both groups using TRIzol Reagent (Thermo Fisher Scientific) following the datasheet of the product. The primer sequences were described in Supplementary Table S4.

For mRNA expression, aliquots of 2 μg of total RNA were reverse transcribed using the High-Capacity Kit RNA-to-cDNA (Life Technologies, 4387406, Carlsbad, CA, USA) in a 10 μL reaction according to the manufacturer’s instructions. Aliquots of cDNA from each sample were added to a mix of reagents containing Power SYBR Green PCR Master Mix (Applied Biosystems, Foster City, CA, USA), primers “sense” and “anti-sense,” and the volume was completed to 10 µL with ultrapure water.

The miRNA RT-qPCRs were performed according to the appropriate literature [87]. The stem-loop RT prime was hybridized with the miRNA molecule, and then a reverse transcription reaction was performed. The reaction products were then amplified using a miRNA-specific forward primer, a universal reverse primer, and a fluorophore from the Power SYBR Green PCR Master Mix (Applied Biosystems, Foster City, CA, USA), and made up to 10 µL with ultrapure water. The RT prime stem-loop, forward and reverse primers were synthesized by the company Thermo Fisher (Waltham, MA, USA) [88].

Reactions were performed in duplicate for each mRNA and miRNA on the Real Time QuantStudio 12K flex System (Applied Biosystems) in 96-well plates following the datasheet product. The relative quantification of each mRNA and miRNA was performed using the 2^−∆∆CT^ method according to Livak et al. (2001) [89]. The values obtained for all samples were normalized using the ratio obtained between the informative gene and the reference Gusb and Gapdh to mRNAs and U6 to miRNA. Values were calculated using the expression ratio of the GLLP/CTR groups.

A Student’s *t* test was applied to test whether the difference was statistically significant between groups, and the difference in expression was considered significant when *p* < 0.05.

### 4.9. Functional Validation of Selected miRNA in a Transfected Human Prostatic Cell Line

To give further insight into the role of selected miRNA networks on prostate cell behavior, the human benign prostate cell line PNT-2 (Cell Bank of Rio de Janeiro, Brazil) was maintained in RPMI 1640 medium with 2 mM L-glutamine, 10% FBS (Fetal Bovine Serum), 50 μg/mL penicillin/streptomycin, and 0.5 μg/mL amphotericin B (GIBCO, Invitrogen, Waltham, MA, USA). Cells were used up to passage 20. The culture medium was changed twice a week throughout the experimental period in a humid atmosphere at 37 °C with 5% CO_2_. An inverted microscope was used to monitor the cells (Zeiss Axiovert, Oberkochen, Germany). Cells were resuspended after trypsin (GIBCO/Invitrogen) treatment for 5 min at 37 °C. In vitro studies were conducted in three independent experiments performed in triplicate.

### 4.10. miRNA Transfection and Cell Viability Assays

The in vitro experiments were divided into three groups. Mimic group: cells transfected to the specific mimic miRNA diluted in lipofectamine (Thermo Fisher, mirVanaTM miRNA Mimic, Catalog # 4464066; Assay ID MC12973); Negative Control group: cells trasnfected with non-specific miRNA diluted in lipofectamine (Thermo Fisher, mirVana TM Mimic, Negative Control 1, # 4464058, Assay AS02D00R); and Lipo Control group: cell exposed only to lipofectamine. Before transfecting, the treatment complex was formed with the Opti-MEM-reduced serum medium (Thermo Fisher Scientific, USA). The PNT-2 cells (8 × 10^4^) and were placed in 24-well plates in 500 μL of complete RPMI medium per well. Once cells became 80% confluent, the RNAiMAX lipofectamine (Thermo Fisher Scientific, USA) was used with or without 10 nM of specific mimic miRNA for 16 was determined h at 37 °C and 5% CO_2_ to perform the transfections. After 24, 48, and 72 h, the cell viability using the MTT reduction method [90] according to the manufacturer’s instructions (Sigma-Aldrich, USA). Absorbance is proportional to cell viability and was quantified in a spectrophotometer (ASYS HITECH GmbH, Eugendorf, Austria) using a 96-well plate at 550 nm absorbance.

### 4.11. Wound Healing Assay

In order to evaluate the potential role of selected miRNA in modulating wound healing capacity in vitro, the PNT-2 cells were cultured in RPMI 1640 medium and plated in 6-well plates. The cells were grown at 37 °C and 5% CO_2_ until they reached 80–100% confluence after transfection. Following that, a vertical single-line scratch was physically created at the highest diameter of each well plate in the identical spot in all replicates using a 200-μL plastic tip. Two PBS washes were used to exclude cell debris, and 1.2 mL of RPMI enriched with 10% FBS was added to each well. The wound open area was captured on camera and evaluated at 0, 24, 48, and 72 h, and the results were presented as percentage wound healing. Time 0 h is the time point immediately after the scratch.

### 4.12. In Vitro and In Silico Validation of Selected miRNA-mRNA Networks

The total RNA of PNT-2 cells from all treatments was extracted with TRIzol (Thermo Fisher Scientific, USA) as recommended by the company. The cDNA synthesis and the RT-qPCRs to mRNAs and miRNAs were conducted as described in chapter 2.6. The primer sequences were shown in Supplementary Table S4. The Human Protein Atlas (HPA) database (https://www.proteinatlas.org/ (accessed on 7 July 2021)) was used for in silico validation of selected targets in both normal and tumor samples from the human prostate.

### 4.13. Statistical Analysis

GraphPad Prism^®^ software was used for statistical analysis (version 5.00, Graph Pad, Inc., San Diego, CA, USA). The data were expressed as mean ± SD, and the results were subjected to a *t* test. Differences were considered statistically significant when *p* < 0.05.

## 5. Conclusions

Here we provide new evidence on the participation of the miR-18a-5p/P4HB network in addition to modulation of estrogen receptors mediating prostate carcinogenesis in maternally malnourished offspring rats. Knowledge of these conditions can help to elucidate the mechanistic framework involved in prostate carcinogenesis and highlight the importance of discussing maternal food security as public policy for the lifelong prevention of chronic diseases in the progeny, as proposed by the DOHaD concept.

## Figures and Tables

**Table 1:** Biometric and metabolic parameters of pregnant and offspring rats from the CTR and GLLP groups.

Parameters	CTR	GLLP	*p*-Value
Pregnant body weight at gestational day 21 (g)	368.00 ± 16.49	343.80 ± 14.49 *	0.004
Pregnant energy intake (Kj/day)	310.30 ± 22.01	326.20 ± 61.38	0.3651
Female offspring BW at PND 1 (g)	7.2 ± 0.52	5.9 ±0.35 *	<0.0001
Male offspring BW at PND 1 (g)	7.367± 0.52	6.307 ± 0.49 *	<0.0001
Number of pups per litter	10.50 ± 1.87	10.29 ± 1.38	0.9264
Litter male/female ratio	0.97 ± 0.21	1.08 ± 0.26	0.1648
Female offspring BW at PND 21 (g)	47.4 ± 4.30	22.3 ± 4.5	<0.0001
Male offspring BW at PND 21 (g)	37.23 ± 6.57	20.03 ± 3.88 *	<0.0001
Absolute VP weight at PND 21 (g)	0.32 ± 0.08	0.14 ± 0.03 *	<0.0001
Relative VP weight at PND 21	0.92 ± 0.19	0.7893 ± 0.16 *	0.0398
Testosterone at PND 21 (ng/mL)	0.72 ± 0.32	1.84 ± 0.77 *	0.0024
Estrogen at PND 21(pg/mL)	18.28 ± 1.15	20.1 ± 3.35 *	0.0177
Male offspring BW at PND 540 (g)	428.30 ± 9.73	376.80 ± 6.34 *	<0.0001
Absolute VP weight at PND 540 (g)	0.62 ± 0.03	0.65 ± 0.04	0.6686
Relative VP weight at PND 540	1.44 ± 0.40	1.73 ± 0.60	0.0512
Testosterone at PND 540 (ng/mL)	2.73 ± 0.38	1.68 ± 0.18 *	<0.0001
Estrogen at PND 540 (pg/mL)	14.64 ± 4.76	35.88 ± 4.42 *	0.0023

All data were expressed as mean ± SD. Dams n = 18/group; offspring (n = 18/group) from each age; offspring hormonal analyses (n = 8/group) from each age. The asterisk represents the statistical differences between experimental groups with *p* < 0.05. CTR: Control Group; GLLP: Gestational and Lactational Low Protein; BW: body weight; VP: ventral prostate; PND: postnatal day.

## Data Availability

The Gene Expression Omnibus (GEO) database data can be found in these accession GSE180674 and GSE180673. The proteomic data can be found in [19].

## Supplementary Materials

The following supporting information can be downloaded at: https://www.mdpi.com/article/10.3390/ijms232314855/s1.
